# Enhancing Classification Accuracy with Integrated Contextual Gate Network: Deep Learning Approach for Functional Near-Infrared Spectroscopy Brain–Computer Interface Application

**DOI:** 10.3390/s24103040

**Published:** 2024-05-10

**Authors:** Jamila Akhter, Noman Naseer, Hammad Nazeer, Haroon Khan, Peyman Mirtaheri

**Affiliations:** 1 Department of Mechatronics and Biomedical Engineering, Air University, Islamabad 44000, Pakistan; jamila.akhter@mail.au.edu.pk (J.A.); hammad@mail.au.edu.pk (H.N.); 2 Department of Mechanical, Electrical, and Chemical Engineering, OsloMet—Oslo Metropolitan University, 0176 Oslo, Norway; haroonkh@oslomet.no (H.K.); peymanm@oslomet.no (P.M.)

**Keywords:** brain–computer interfacing (BCI), functional near-infrared spectroscopy (fNIRS), long short-term memory (LSTM), bidirectional long short-term memory (Bi-LSTM), integrated contextual gate network (ICGN), deep learning (DL)

## Abstract

Brain–computer interface (BCI) systems include signal acquisition, preprocessing, feature extraction, classification, and an application phase. In fNIRS-BCI systems, deep learning (DL) algorithms play a crucial role in enhancing accuracy. Unlike traditional machine learning (ML) classifiers, DL algorithms eliminate the need for manual feature extraction. DL neural networks automatically extract hidden patterns/features within a dataset to classify the data. In this study, a hand-gripping (closing and opening) two-class motor activity dataset from twenty healthy participants is acquired, and an integrated contextual gate network (ICGN) algorithm (proposed) is applied to that dataset to enhance the classification accuracy. The proposed algorithm extracts the features from the filtered data and generates the patterns based on the information from the previous cells within the network. Accordingly, classification is performed based on the similar generated patterns within the dataset. The accuracy of the proposed algorithm is compared with the long short-term memory (LSTM) and bidirectional long short-term memory (Bi-LSTM). The proposed ICGN algorithm yielded a classification accuracy of 91.23 ± 1.60%, which is significantly (*p* < 0.025) higher than the 84.89 ± 3.91 and 88.82 ± 1.96 achieved by LSTM and Bi-LSTM, respectively. An open access, three-class (right- and left-hand finger tapping and dominant foot tapping) dataset of 30 subjects is used to validate the proposed algorithm. The results show that ICGN can be efficiently used for the classification of two- and three-class problems in fNIRS-based BCI applications.

## 1. Introduction

Brain–computer interface (BCI) technology provides direct communication between the user’s brain and actuation devices. Initially, the intention to use BCI technology was to design assistive devices for biomedical applications and to restore the movement ability of paralyzed and severely handicapped individuals to gain motor functionality [1]. In recent times, future predictions for BCI have motivated researchers to decode the motor activities of non-paralyzed individuals to control the systems without any use of physical manpower [2]. BCI systems acquire signals, i.e., perception, and communicate with the physical environment, i.e., control external devices such as the exoskeleton or wheelchair [3]. The challenging factors in BCI are acquiring signals with high quality, and preprocessing and classifying them to generate commands for the control of external devices [1]. To know the motor intentions of humans to control Internet of Medical Things technology, external rehabilitation, and prosthetic devices, researchers need to know how and from where motor signals can be acquired.

Until now, to record the motor activity of humans, modalities such as electroencephalography (EEG) [4,5,6,7,8], functional magnetic resonance imaging (fMRI) [9,10], positron emission tomography (PET), and functional near-infrared spectroscopy (fNIRS) [10,11,12,13] have been introduced, along with their decoding algorithms. EEG is designed to acquire a complex set of signals from the brain based on the potential difference created by neuronal signal conduction in the brain. fMRI uses the magnetic resonance imaging technique and fNIRS uses the near-infrared technique to acquire brain signals. Over the last two decades, fNIRS has become a well-known neuroimaging modality to capture cortical tissue’s hemodynamic response, with high spatial resolution [14,15]. Comparing EEG and fMRI, fNIRS is less sensitive to motion artifacts and captures brain activity signals with less complexity. Still, fNIRS faces challenges in experimental setups due to deviations in statistical results [16,17].

The current trend in fNIRS-based BCI applications is to improve signal quality by processing the acquired brain signals. For this purpose, extracting the features from fNIRS signals manually or by applying the available feature extraction techniques, such as z-score [18], non-linear feature extraction [19], and statistical features [20], requires a deep knowledge of the investigated neuro-physiological phenomenon to extract these features. Also, the computational cost increases in feature extraction for classification through machine learning (ML) classifiers (support vector machine (SVM), k-nearest neighbor (KNN), linear discriminant analysis (LDA), decision tree, etc.). Recently, new approaches have been introduced in deep learning (DL) algorithms [20,21], substituting manual feature extraction and then classification using ML. The DL algorithm overcomes these challenges in feature extraction and selection for a specific activity and provides promising dedication for data preprocessing, real-time feature extraction, and classification to generate control commands for BCI applications [22,23]. DL algorithms effectively learn latent correlations and can extract more discriminative features from filtered datasets with higher computational speed. For feature extraction from the filtered fNIRS dataset, recurrent neural network (RNN) algorithms can capture desired patterns and temporal features from time series data over a long period [24]. RNN algorithms like long short-term memory (LSTM), bidirectional long short-term memory (Bi-LSTM), and gated recurrent units (GRU) are used for feature extraction and the classification of complex sequential tasks. However, a vanishing gradient during backpropagation limits LSTM’s capacity to learn and retain information over extended periods. The vanishing gradient problem impacts LSTM’s performance in long-term dependency tasks [25]. The available methods to cope with the vanishing gradient problem are the careful initialization of weights [26] and gradient clipping that sets a threshold for the gradients during training [27].

The weight initialization choice depends on network depth, activation functions, and specific task requirements and is assigned on a random basis [26]. Gradient clipping involves a set threshold, beyond which gradients are scaled during backpropagation, where the threshold values depend upon model architectures, data distribution, and datasets [28]. GRU performs comparably or even outperforms LSTMs; however, in more challenging and complex scenarios with intrinsic dependencies, the simplicity of GRU becomes a limitation [29].

This study proposes an integrated contextual gate network (ICGN) as a DL algorithm. The suggested algorithm contains cells that integrate input data, previous cell state, and previous hidden state to generate the gating outputs and cell state. ICGN cell consists of gate mechanisms that regulate the flow of information, similar to the LSTM input: forget and output gates. In ICGN, the memory cell is proposed, which is regulated by the cell’s internal state and outputs from all three gates. ICGN implies a neural network architecture where input data, previous cell state, and previous hidden state are all crucial in determining the gating outputs and internal cell state, ultimately contributing to the final cell state computation.

## 2. Materials and Methods

This study involves data collection from the motor cortex during the hand-gripping motor activity. Acquired data are preprocessed to remove artifacts and enhance raw data quality and the proposed ICGN model is applied for classification.

### 2.1. Participants

The fNIRS-based hemodynamic dataset was collected in the fNIRS neurorobotics research lab at Air University, Islamabad. Data collection was approved by the Institutional Review Board of Air University, Islamabad, Pakistan (approval number: AU/EA/2022/02/011). The experimental data collection follows guidelines provided by the most recent version of the Declaration of Helsinki, and informed consent was obtained from all the participants. A total of twenty right-handed participants (10 male and 10 female) were recruited for the experimental study. The participants’ ages fall within a range of 20 ± 5 years. All participants were rigorously screened to ensure the absence of any neurological conditions. Additionally, they refrained from consuming caffeine for a minimum of four hours leading up to the data collection phase of the experiment.

### 2.2. Experimental Paradigm/Protocol

**Figure 1:**
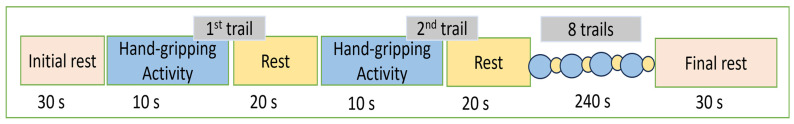
A paradigm for experimental data collection. The total duration per subject is 360 s, with 30 s initial and 30 s end rest intervals separated by 10 trials of 10 s activities and 20 s rest intervals.

The experimental paradigm for data acquisition is depicted in Figure 1. The experiment started with a 30 s initial rest followed by ten trials of 10 s activity and 20 s rest and ended with an additional 30 s rest. The total duration of the experiment for each participant is 360 s.

### 2.3. Experimental Setup

**Figure 2:**
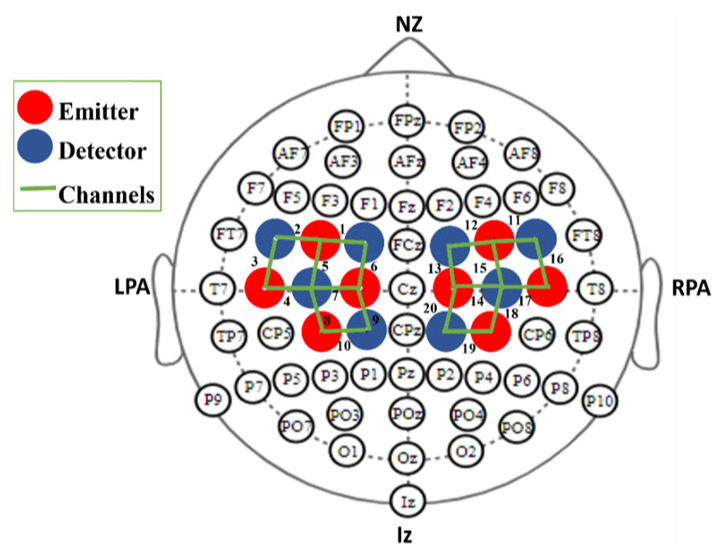
The optodes are positioned on the motor cortex according to the 10–20 international system. Red and blue circles denote emitters and detectors, respectively. Green lines represent channels along with numbers. A configuration of eight emitters and eight detectors spaced 3 cm apart resulted in twenty channels in total.

A continuous-wave imaging system (NIRSport2 data acquisition system by NIRx medical technologies (Germany) was used to acquire fNIRS data. Eight emitters and eight detectors were positioned over the motor cortex with a separation of 3 cm [30] according to the 10–20 standard system as shown in Figure 2. Twenty channels were created by the arrangement of optodes (emitter and detectors pair) on the expected motor cortex region.

### 2.4. Signal Acquisition and Processing

NIRSport2 is an fNIRS data acquisition system, which is equipped with two wavelengths: 760 and 850 nm. For experimental data collection, the sampling frequency was set to 10.1725 Hz. Modified Beer-Lambert law (MBLL) was used to calculate hemodynamic response function where the light intensity sensed by optodes on the scalp surface was converted into a change in oxyhemoglobin (ΔHbO) and deoxyhemoglobin (ΔHbR) concentrations using Equation (1) by MBLL [31].

(1) $$\left[\begin{array}{c} \Delta HbO\left(t\right)\\ \Delta HbR\left(t\right) \end{array}\right]=\frac{{\left[\begin{array}{cc} {Ɛ}_{HbO}\left(\lambda_{1}\right) & {Ɛ}_{HbR}\left(\lambda_{1}\right)\\ {Ɛ}_{HbO}\left(\lambda_{2}\right) & {Ɛ}_{HbR}\left(\lambda_{2}\right) \end{array}\right]}^{-1}\left[\begin{array}{c} \Delta A\left(t;\lambda_{1}\right)\\ \Delta A\left(t;\lambda_{2}\right) \end{array}\right]}{l\times d}$$

where

➢ ΔHbO(t), ΔHbR(t) are the concentration changes in [μM];
➢ ${Ɛ}_{HbO}\left(\lambda \right),\;{Ɛ}_{HbR}\left(\lambda \right)$ are the extinction coefficients of HbO and HbR in $\left[\mu M^{-1}cm^{-1}\right]$;
➢ $A\left(t;\lambda ₁\right),\;A\left(t;\lambda_{2}\right)$ are the absorbance measured at time t using two different wavelengths $\lambda_{1}$ and $\lambda_{2}$;
➢ l = distance between source and detector (3 cm);
➢ d = differential path length factor.

**Figure 3:**
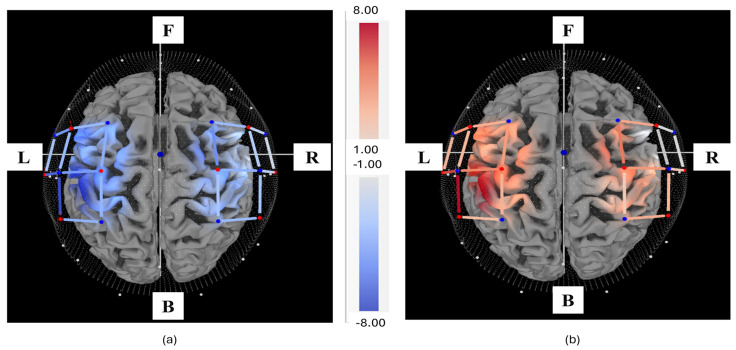
Top view of the topographical map for the hand-gripping activity and rest, where F, B, L, and R are front, back, left, and right sides, respectively. The activity and rest values are represented as changes in the concentration of oxyhemoglobin (μM). (**a**) Rest and (**b**) Activity.

A band-pass filter with a passband of 0.01–0.2 Hz was used to eliminate motion artifacts and instrumental and physiological noises. The filtered signal was transformed into ΔHbO and ΔHbR concentrations for each subject using Equation (1). The ΔHbO concentration for each subject consists of a total of 3661 samples (360 s of data). This includes 610 samples (baseline samples) from both the initial and final 30 s of rest, 1017 samples from 100 s of activity across 10 trials, and 2034 samples from 200 s of rest across 10 trials. The topographical maps plotted using average activity values (1017 samples) and average rest values (2034 samples) are represented in Figure 3a and Figure 3b, respectively. Figure 3 was plotted using Satori software (version 2.0, NIRx Medical Technologies), where activity and rest values are represented in the form of change in the concentration of oxyhemoglobin (ΔHbO) measured in μM.

Once the data were converted into the ΔHbO and ΔHbR concentrations, the data were labeled. To label the data, the paradigm in Figure 1 was used, where the initial and final rest (baseline samples) were excluded. Rest during 10 trials of activity performance was labeled as class 1, and activity trials were labeled as class 2; then, after labeling, the data were split into an 80% training set, a 10% validation set, and a 10% testing dataset, where an equal number of samples from class 1 and 2 were picked randomly. In this way, data samples for training, testing, and validation were picked from each trial of activity and rest. These training, testing, and validation datasets were then used to train, validate, and test the classification algorithms discussed in the next section as input values (xₜ). A two-tailed t-test was conducted to compare the means of the activity and rest classes. The results show a statistically significant difference between the data classes, with a *p*-value of 0.001 and a t-value of 3.695.

### 2.5. Signal Classification

In BCI applications, features are sometimes manually extracted from datasets to use ML classifiers for classification. In this study, DL algorithms are selected for the classification, which extract features based on patterns within datasets. In the following explanation, LSTM, Bi-LSTM, and the proposed ICGN algorithms are discussed.

#### 2.5.1. LSTM

**Figure 4:**
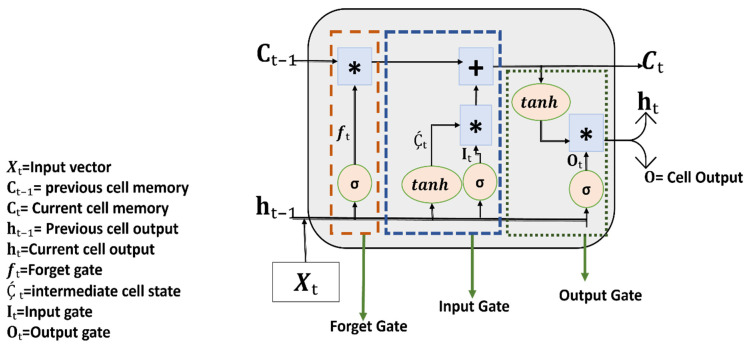
Illustration of the LSTM cell architecture, showcasing its internal mechanisms for capturing and retaining sequential information, including the input, forget, and output gates, as well as the cell state, hidden state, and various activation functions. Pink color circles represent sigmoid functions, pink color oval shapes represent hyperbolic functions, blue square shape boxes with * symbol represent multiplication sign and dashed lines boxes represent gates.

LSTM is an RNN algorithm and introduces gate mechanisms: forget gate, input gate, and output gate, given in Equations (2)–(4). These are used to filter irrelevant information [32]. The sigmoid function for activating these gates assigns 0 and 1 values. In the forget gate, 0 value is to discard the features from the network and 1 signifies that the network should store feature value to update the cell state. The input gate determines and computes new values to update the cell state and the output gate determines which cell states and inputs to the current unit are relevant to the output. The LSTM cell gates depend on the current input value (xₜ), the past hidden state (ht − 1), and biased values (bf, bi, and bo) [33]. Equation (5) presents the candidate cell state (Ḉt) containing new information and passes it to the cell state, depending on the current input value (xₜ) and past hidden state (ht − 1). The activation function for the candidate cell state is tanh, so new information about the cell state is between −1 and 1. If the Ḉt value is negative, new information is subtracted from the cell state when its positive information is added to the cell state at time t. In Equation (6), Ct represents the cell state, and it is the product of the output from the forget gate and the previous cell state which sums up with the product of the output from the input gate and candidate cell state. The cell state represents the memory of the complete LSTM network and brings information about the entire sequence. Equation (7) represents the LSTM hidden state, which depends upon a product of the output gate values and the tangent hyperbolic of cell state [34]. The LSTM cell is shown in Figure 4.

*F_t_* = *σ* (*wₓ_f_xₜ* + *w_hf_h_t_*_−1_+ *b_f_*) (2)

*I_t_* = *σ* (*wₓᵢxₜ* + *w_hi_h_t_*_−1_ + *b_i_*) (3)

*O_t_* = *σ* (*wₓoxₜ* + *w_ho_h_t_*_−1_ + *b_o_*) (4)

*Ḉ_t_* = *tanh* (*wₓ_f_xₜ* + *w_hf_h_t_*_−1_ + *b_f_*) (5)

*C_t_* = *F_t_ₓ* × *C_t_*_−1_+ *I_t_ₓ* × *Ḉ_t_* (6)

*H_t_* = *O_t_ tanh* (*C_t_*) (7)

where

It, Ft, and Ot = output from the input, forget, and output gates;
σ = sigmoid activation function;
tanh = hyperbolic tangent activation function;
wₓᵢ, wₓf, and wₓo = weight functions of input forget, and output gates;
xₜ = input values at time t;
ht−1 = previous cell hidden state;
Ct−1 = previous cell state;
Ḉt = internal cell state;
bi, bf, and bo = biased values of the input, forget, and output gates;
Ct = memory cell;
Ht = hidden state.

#### 2.5.2. ICGN

To cope with the long-term dependencies and vanishing gradient limitation in LSTM, an ICGN cell, along with an algorithm, is proposed. In the ICGN cell, three gates and an internal cell state similar to those in LSTM are introduced. Input, forget, and output gates are sigmoid activation functions of the current input values, previous hidden state, previous cell state, and biased values presented in Equations (8)–(10), respectively. The internal cell state is a hyperbolic tangent activation function of the current input values, previous hidden state, and previous cell state presented in Equation (11). The memory cell introduced in the ICGN cell given in Equation (12) depends on the internal cell state and output from the input, forget, and output gates. The hidden state of the ICGN cell given in Equation (13) is a product of output gate information and the hyperbolic tangent activation function of the memory cell of ICGN.

*I_t_* = *σ*(*wₓᵢxₜ* + *w_hi_h_t_*_−1_ + ***w_ci_c_t_*_−1_**_+_
*b_i_*) (8)

*F_t_* = *σ*(*wₓ_f_xₜ* + *w_hf_h_t_*_−1_ + ***w_cf_c_t_*_−1_**_+_
*b_f_*) (9)

*O_t_* = *σ*(*wₓoxₜ* + *w_ho_h_t_*_−1_ + ***w_cf_c_t_*_−1_**_+_
*b_o_*) (10)

*Ḉ_t_* = *tanh*(*wₓ_f_xₜ* + *w_hf_h_t_*_−1_ + ***w_cf_c_t_*_−1_**_+_
*b_f_*) (11)

***C_t_* = *F_t_* × *Ḉ_t_* + *I_t_* × *Ḉ_t_* + *O_t_* × *Ḉ_t_*** (12)

*H_t_* = *O_t_ tanh* (*C_t_*) (13)

**Figure 5:**
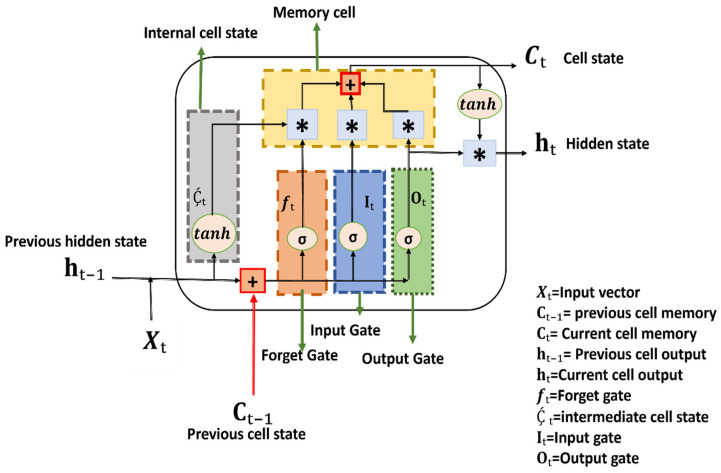
The proposed ICGN cell’s input, forget, and output gates depend upon the previous cell state, previous hidden state, and current input values, while internal cell states depend on the previous hidden state and current input values only. Information from all three gates and internal cell states is summed and evaluated in the memory cell, where the final cell state is generated. Pink color circles represent sigmoid functions, pink color oval shapes represent hyperbolic functions, blue square shape boxes with * symbol represent multiplication, red square shape boxes with + symbol represent addition and dashed lines boxes represent gates.

The ICGN cell is shown in Figure 5.

#### 2.5.3. Bi-LSTM

**Figure 6:**
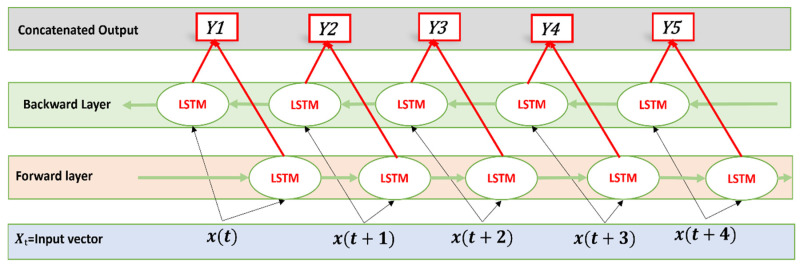
Bi-LSTM, contains a forward layer and a backward layer. Output from both layers is concatenated to yield the final output. Arrows represent the direction of the flow of information between Bi-LSTM layers.

The bidirectional nature of the Bi-LSTM allows it to process the input sequence both in the forward and backward directions. Bi-LSTM is adept at capturing contextual information from past as well as future time steps and effectively captures dependencies in sequential data. The Bi-LSTM algorithm contains 128 units in the first Bi-LSTM layer. Following this, a dropout layer of 0.1 is added. Finally, two dense layers are used: in the first dense layer there are 64 units with RelU activation function, and in the second dense layer, 2 units and the sigmoid activation function are used. The architecture of the Bi-LSTM is given in Figure 6.

## 3. Results

In this study, three DL classification algorithms (LSTM, Proposed ICGN, and Bi-LSTM) are trained and tested on two classes (activity and rest) of hand-gripping fNIRS data from twenty subjects. Validation of the proposed algorithm is performed on three classes (right- and left-hand finger tapping, and dominant foot tapping) from open access datasets [35]. Each subject’s data (in both the two-class and three-class datasets) is split into training, testing, and validation sets separately, with 80% of the data used for training, 10% for validation, and 10% for testing. This process is performed individually for each subject. In this section, the two-class and three-class dataset results are presented to compare the proposed DL algorithm with the LSTM and Bi-LSTM algorithms.

### Two-Class Dataset Results

**Figure 7:**
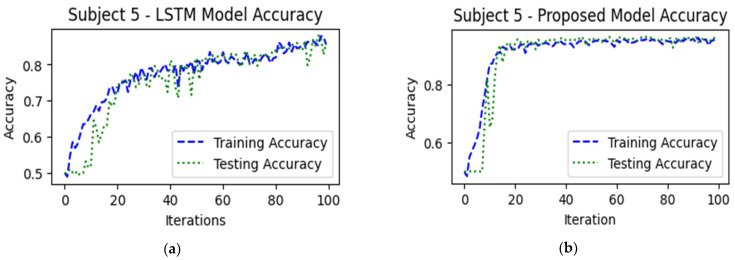

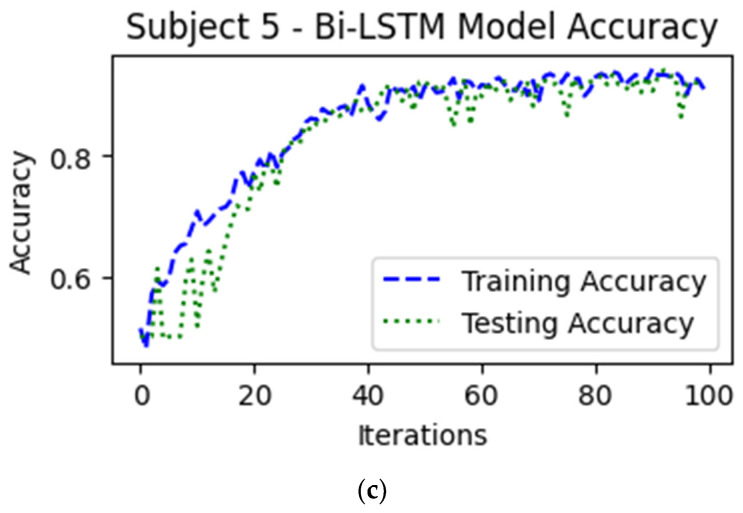
This figure illustrates the algorithms’ training and testing accuracies and represents the performance of (**a**) LSTM’s performance during training and testing, (**b**) proposed ICGN algorithm’s performance during training and testing, and (**c**) Bi-LSTM’s performance during training and testing with the 2-class hand-gripping fNIRS data (HbO).

The training and testing accuracies presented in Figure 7 illustrate the performance of the LSTM, proposed ICGN, and Bi-LSTM algorithms over successive training iterations. Notably, the proposed ICGN model demonstrates early convergence in both training and testing accuracies compared to LSTM and Bi-LSTM, indicating efficient learning within fewer training iterations. This type of behavior highlights how well the proposed ICGN cell and algorithm capture intricate patterns and relationships in the data without overfitting.

**Figure 8:**
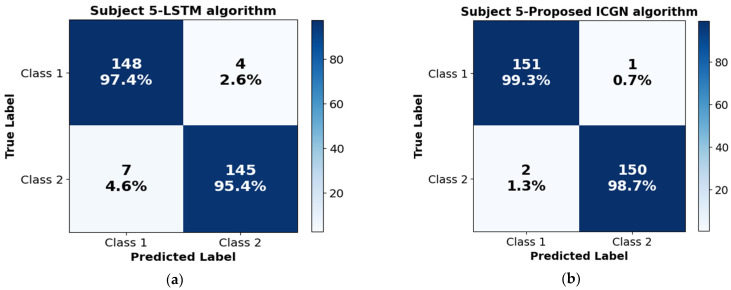

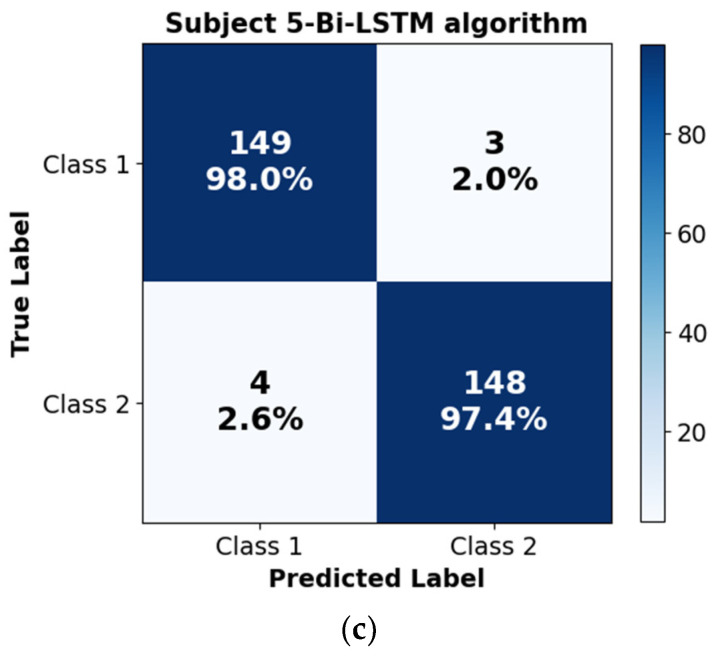
This figure illustrates the confusion matrices representing the performance of (**a**) LSTM algorithm’ performance confusion matrix, (**b**) proposed ICGN algorithm performance confusion matrix, and (**c**) Bi-LSTM algorithm performance confusion matrix trained with 2-class hand-gripping fNIRS data: HBO. Each matrix provides insights into the algorithm’s ability to correctly classify the instances, presenting a visualization of true-positive, false-positive, true-negative, and false-negative counts.

Employing the confusion matrix in Figure 8 emphasizes evaluating the precision and inaccuracy patterns of the LSTM, proposed ICGN, and Bi-LSTM algorithms. Specifically, the algorithm’s capacity to accurately categorize positive and negative predictions is summarized in the confusion matrix. The significantly greater counts of true positives (TPs), 151 samples, and true negatives (TNs), 150 samples, indicate the better efficacy of the proposed ICGN algorithm. These results imply that in comparison to LSTM and Bi-LSTM, the proposed ICGN algorithm might provide improved classification accuracy and consistency throughout a wide variety of classification tasks.

The classification accuracy performances of the LSTM, proposed ICGN, and Bi-LSTM algorithms over a range of subjects are given in Table 1. The average classification accuracy of each algorithm is shown together with the deviation values from the average accuracy values. Notably, for all subjects, the proposed ICGN algorithm consistently performs better in classification accuracy than the LSTM and Bi-LSTM algorithms. It also shows reduced deviation values from the average accuracy, which suggests more constant and reliable performance across many subjects. These results demonstrate the better accuracy, with minimum variance, that the proposed ICGN algorithm obtains during training, which makes it a strong and dependable option for classification tasks for fNIRS-based BCI applications.

**Figure 9:**
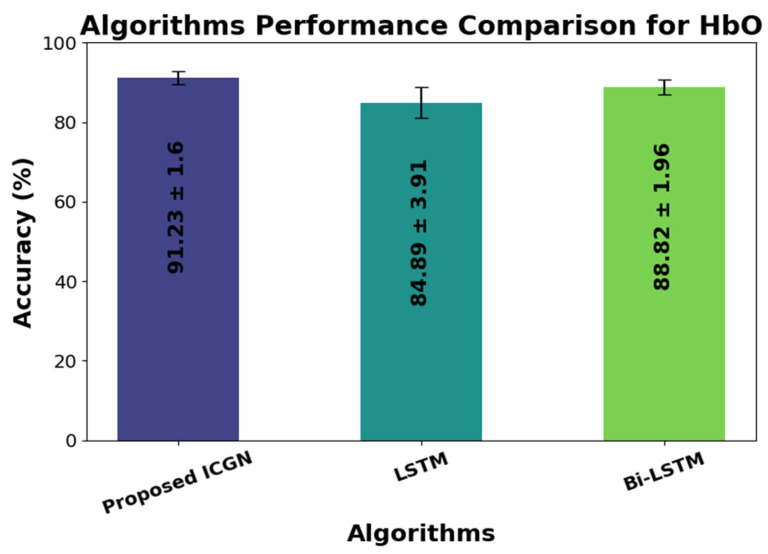
Average classification accuracy performances of the LSTM, proposed ICGN, and Bi-LSTM algorithms for 2-class.

An analysis of the classification accuracy performances of LSTM, the proposed ICGN, and Bi-LSTM is depicted in Figure 9. The bar graph presents a comparative examination of the accuracy values attained by these algorithms. Notably, with a significantly high accuracy rate of 91.23 ± 1.60%, the ICGN algorithm continuously outperformed the LSTM and Bi-LSTM classifiers. These results emphasize how well the proposed ICGN can capture complicated patterns in the fNIRS data.

**Figure 10:**
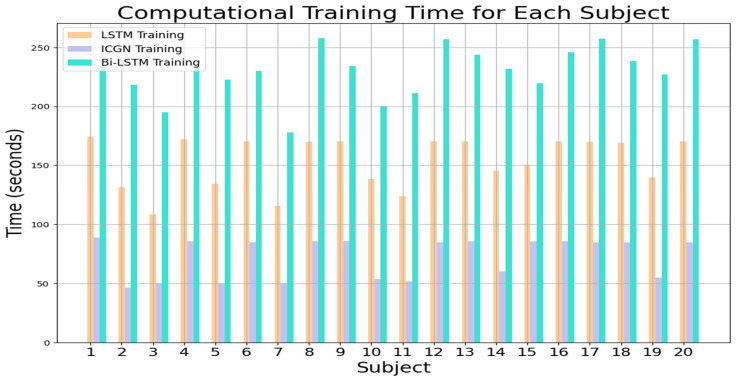
LSTM, ICGN, and Bi-LSTM algorithms’ average training time required for each subject for 2-class dataset.

**Figure 11:**
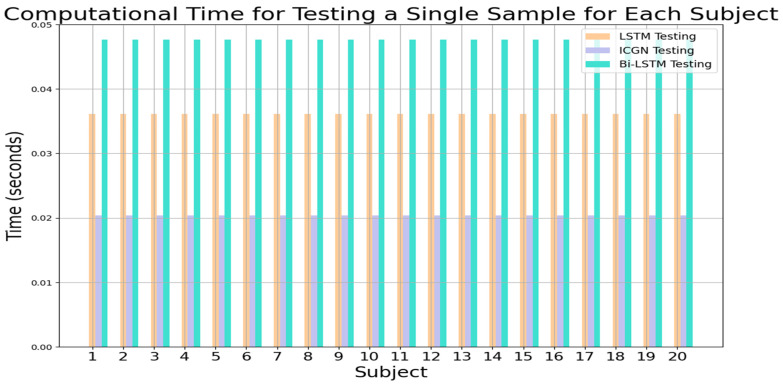
LSTM, ICGN, and Bi-LSTM algorithms’ testing time for a single sample for each subject for 2-class data.

The computational cost of the models is assessed in terms of training and testing time, as shown in Figure 10 and Figure 11, respectively. Training time refers to the time required for the model to train on each subject’s dataset, which includes input data processing, weight optimization, and model convergence. In contrast, testing time indicates the time it takes for the model to make predictions on a single sample of data for each subject, revealing the inference time required for the model to process unknown data. These computational cost analyses shed light on the efficiency and scalability of the proposed model in real-world applications, where time limitations may impact model deployment and usability.

## 4. Validation of Proposed Method on Three-Class Open Access Dataset

The validation of the proposed ICGN algorithm is assessed through the open access fNIRS dataset [35]. This dataset consists of recordings from thirty subjects engaged in motor tasks, including left0 and right-hand finger tapping and dominant foot tapping. Each task consists of 25 trials, providing a comprehensive basis for the performance evaluation of the proposed ICGN algorithm across various motor tasks and subjects. The average classification accuracy yielded by using the ICGN to classify the ∆HbO signal of this dataset is 92.37 ± 7.17%. In comparison, LSTM and Bi-LSTM yield average classification accuracies of 86.20 ± 6.21% and 88.07 ± 5.90%, respectively. The results achieved using the ICGN algorithm have significantly (*p*-value < 0.025) better performance as compared to the results of the LSTM and Bi-LSTM for three-class fNIRS-based BCI problems. The following gives these results in detail.

**Figure 12:**
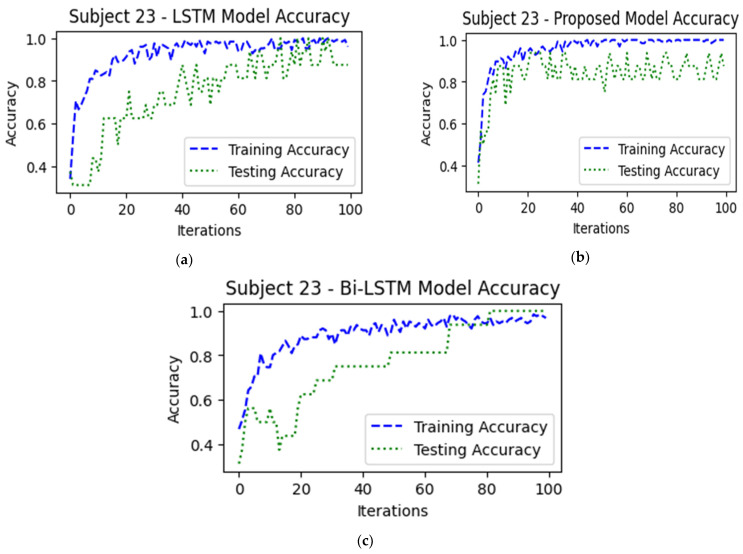
This figure illustrates the algorithms’ training and testing accuracies and represents the performance of (**a**) LSTM during training and testing, (**b**) proposed ICGN algorithm during training and testing, and (**c**) Bi-LSTM during training and testing with 3-class open access fNIRS data (HbO).

**Figure 13:**
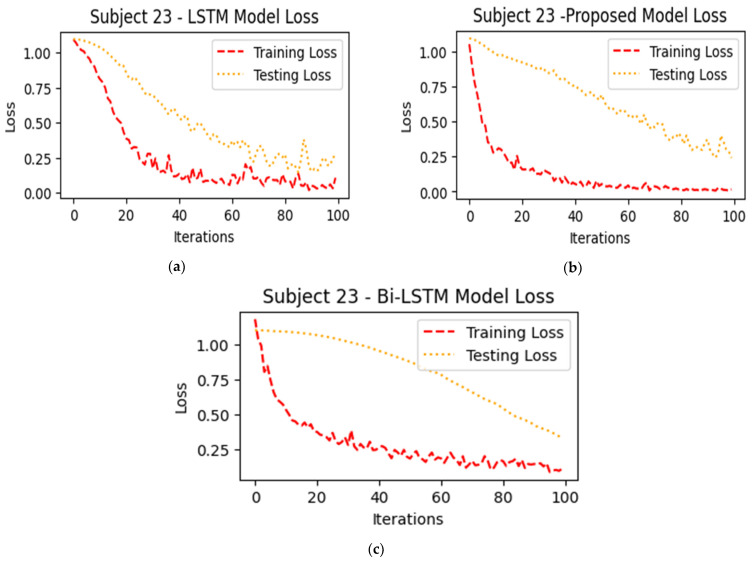
This figure illustrates the algorithms’ losses and represents the performance of (**a**) LSTM, (**b**) proposed ICGN, and (**c**) Bi-LSTM algorithms trained with 3-class open access fNIRS data (HbO).

In Figure 12 and Figure 13, the performances of the LSTM, BiLSTM, and ICGN deep learning algorithms are presented in the form of training and testing accuracies and losses, respectively. In a three-class classification problem, the complexity increases, potentially resulting in higher losses due to the added difficulty of distinguishing between multiple classes, but comparatively, for 100 iterations (the model iterates through the entire training dataset 100 times during the training process), losses decreased to 0.25 in the proposed ICGN algorithm.

**Figure 14:**
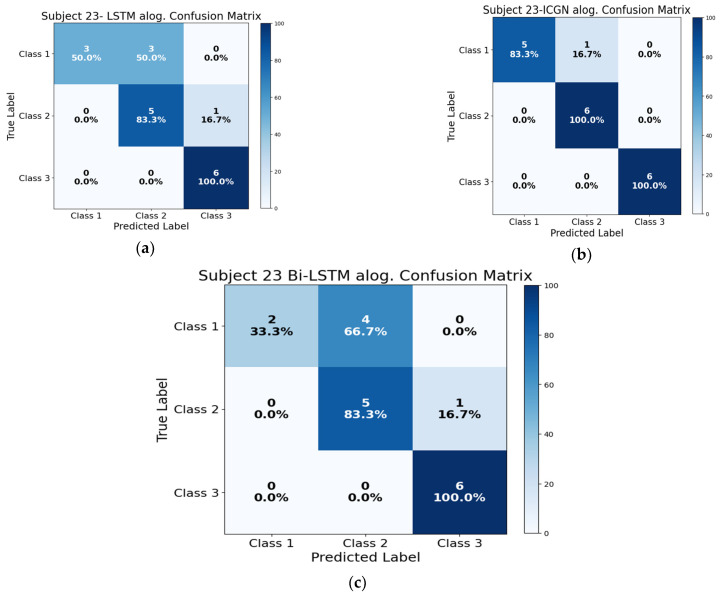
This figure illustrates the (**a**) LSTM algorithm performance confusion matrix, (**b**) proposed ICGN algorithm performance confusion matrix, and (**c**) Bi-LSTM algorithm performance confusion matrix when trained with 3-class open access fNIRS data: HBO.

The LSTM, ICGN, and Bi-LSTM algorithms’ ability to correctly classify the instances of a subject is presented in Figure 14. To test the trained models, six samples from each class were used (these samples were excluded during the model training). Figure 14b presents the ICGN algorithm’s test results for subject 23 only, where, in the first row of the confusion matrices, the algorithm correctly predicted five instances of the first class, incorrectly predicted one instance as the second class, and made no errors in predicting instances of the third class. In the second row, the model correctly predicted six instances of the second class and made no errors in predicting instances of the other classes. In the third row, the model correctly predicted six instances of the third class and made no errors in predicting instances of the other classes.

**Figure 15:**
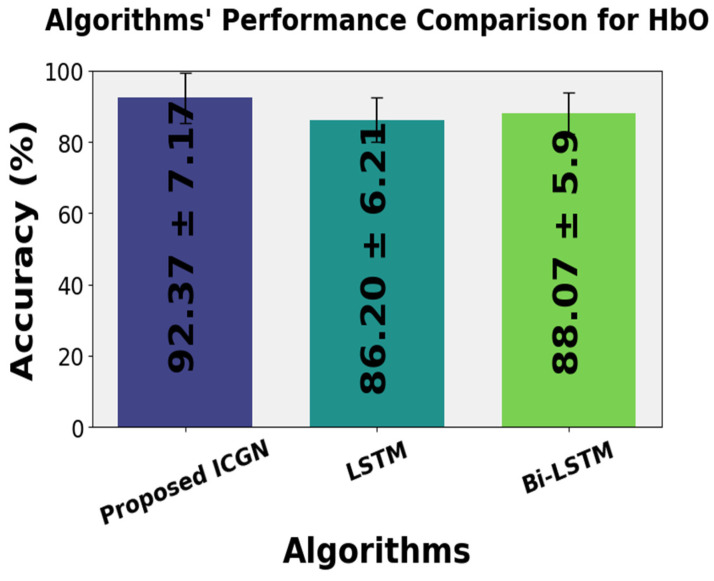
Average classification accuracy of the LSTM, proposed ICGN, and Bi-LSTM algorithms for 3-class.

The average classification accuracy performances of the proposed ICGN, LSTM, and Bi-LSTM for thirty subjects is presented in Figure 15. The comparative accuracy values attained by the ICGN, LSTM, and Bi-LSTM algorithms are 92.37 ± 7.17%, 86.20 ± 6.21, and 88.07 ± 5.9, respectively. These results emphasize the better performance of the proposed ICGN algorithm.

**Figure 16:**
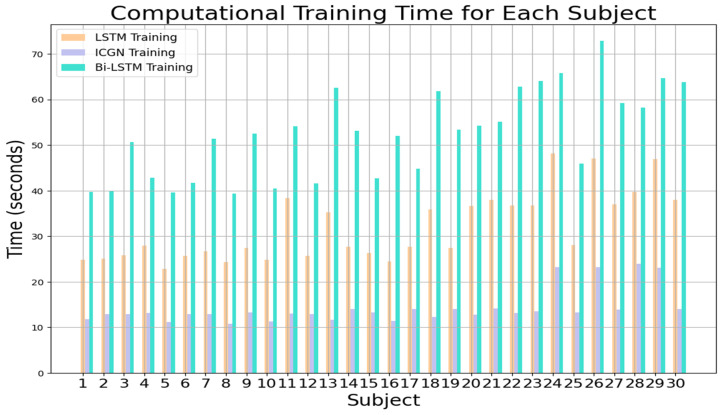
LSTM, ICGN, and Bi-LSTM algorithms’ average training time required for each subject for 3-class data.

**Figure 17:**
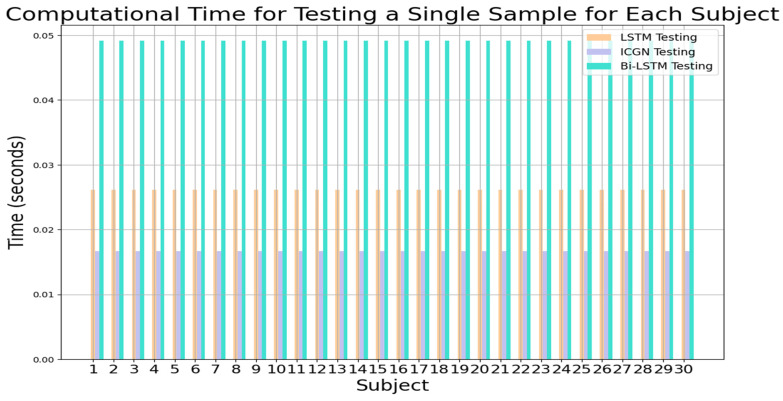
LSTM, ICGN, and Bi-LSTM algorithms’ testing time for a single sample for each subject for 3-class data.

The computational times for training and testing the LSTM, proposed ICGN, and Bi-LSTM with the three-class open access dataset are plotted in Figure 16 and Figure 17, respectively. To test a single subject using the ICGN algorithm, only 0.0125 s is required, which is considerably less than the time required by both the LSTM and Bi-LSTM algorithms.

## 5. Statistical Analysis

An ANOVA test was conducted to assess the statistical significance of the proposed algorithm compared to LSTM and BiLSTM in terms of accuracy for the two-class and three-class datasets. The results of the ANOVA test for the two-class dataset are as follows: F-statistic: 28.731; *p*-value: 2.346 × 10^−9^. Additionally, the post hoc Tukey HSD test was performed to determine specific differences between the groups, presented in Table 2.

The results of the ANOVA test for the three-class dataset are as follows: F-statistic: 17.885; *p*-value: 3.115 × 10^−7^. The post hoc Tukey HSD test results are presented in Table 3.

These results in Table 2 and Table 3 indicate significant differences in accuracy between all pairs of groups: BiLSTM vs. LSTM, BiLSTM vs. proposed ICGN algo., and LSTM vs. proposed ICGN algo. (*p* < 0.05). Therefore, the null hypothesis that there is no difference in accuracy between the groups is rejected in all cases.

## 6. Discussion

In this study, the authors proposed a new deep learning algorithm, ICGN, to increase fNIRS-BCI performance, specifically in terms of classification accuracy and computational cost. In the literature, the latest studies have also focused on improving the classification accuracies of fNIRS-BCI systems by deep learning classification techniques [33,36,37]. Precision, consistency, and less computational power consumption in fNIRS-based BCI could lead to several useful applications in neurorobotics, neuroergonomics, and rehabilitation.

In the past, bundles of studies have been performed to improve the accuracy of fNIRS-based BCI applications. Huma Hamid et al. [38] presented a study to compare the ML classifiers (SVM, K-NN, and LDA) with DL (CNN, LSTM, and Bi-LSTM) algorithms to perform the classification of two classes of walk and rest tasks and reported better performance for DL algorithms. A similar study presented by Mahmudul Haque Milu et al. [39] applied ML (support vector machine (SVM) and linear discriminant analysis (LDA)) and compared it with CNN; they reported that CNN performed well in automatic feature extraction as compared to ML. The conventional LSTM algorithm classifies signals based on the input, forget, and output gate mechanism. Previous cell information in the network is incorporated only for a short period, which makes it suitable only for simple pattern signals. Previous cell information in the LSTM network is forgotten after a few cells, which decreases LSTM’s capability to remember and use previous information to predict future patterns in the dataset. Due to this drawback in LSTM, the concept of decision fusion, which combines DL algorithm outputs, is applied for precise and accurate classification prediction and has obtained improved performance [33] with increased computational cost. Previous studies using LSTM in combination with CNN and other DL neural networks [40,41], where CNN and LSTM are combined to extract the features from complex brain patterns for more precise classification, obtained enhanced performance of fusion of DL models at the cost of increased processing and computational time. Fernandez Rojas et al. [42] present a hybrid CNN-LSTM model with an accuracy of 91.2 ± 11.7, compared to 86.4 ± 16.8 and 88.4 ± 21.1 for the CNN and LSTM models, respectively. Md. Hasin Raihan Rabbani and Sheikh Md. Rabiul Islam [33] introduced a CNN–LSTM–GRU model for EEG and fNIRS fusion with 96% classification accuracy. They also observed an increase in computational cost with the combined model of deep learning algorithms. The temporal convolutional network (TCN) model [43] achieved 85.63% (HbO) and 86.21% (HbR) accuracy in the MI task, and 96.84% (HbO) and 94.83% (HbR) accuracy in the MA task.

In the present study, a deep learning algorithm, ICGN, is proposed to reduce the computational cost with the concept of a decision-fused DL model. The performance improvement in the ICGN algorithm is due to the focus on the previous cell information in the DL neural network. The ICGN algorithm is the DL neural network in which the current cell in a layer works on information from the previous cells’ hidden state, cell state, and current input values. Along with the forget, input, and output gates, an internal cell state is created, which combines the decisions from all three gates and generates an output from the ICGN cell for the next cell in the ICGN neural network. Due to previous cell dependency in the proposed ICGN neural network, complex fNIRS activity patterns/features are more accurately extracted for the classification of the complex tasks as compared to LSTM.

Furthermore, information from the internal cell state of the proposed ICGN cells is summed up with the information from all three gates and current input values to generate the current cell state and hidden state. In this way, each cell in the network keeps information over a long period during feature extraction and pattern generation, which plays a vital role in enhancing the classification accuracy of fNIRS-based BCI applications. The ICGN algorithm achieves improved classification accuracy for fNIRS signals by employing an enhanced feature extraction method and leveraging the inter-dependency among network cells, resulting in a noticeable reduction in computational and processing costs. However, the performance of the model is affected by the different values of the parameters selected for the ICGN algorithm; when considering the number of neurons in the ICGN layer, it is detected that increasing the number from 64 to 128 generally leads to a decrease in average accuracy across different learning rates, dropout rates, and loss functions. This suggests that a more complex model does not necessarily translate to improved performance and may even result in overfitting. Secondly, the dropout rate plays an important role in model performance. For instance, with 64 neurons and a dropout rate of 0.1, the average accuracy ranges from 50.23% to 85.84% for different learning rates and loss functions. However, when the dropout rate is increased to 0.2, there is a notable improvement in average accuracy, with values ranging from 55.71% to 94.3%. This indicates that regularization techniques such as dropout can effectively prevent overfitting and enhance model generalization. Thirdly, the choice of learning rate and loss function significantly impacts model convergence and performance. Lower learning rates (0.001) generally yield higher average accuracies compared to learning rates (0.010), regardless of the dropout rate and number of neurons. Additionally, the choice between the categorical_crossentropy and mean_squared_error loss functions also influences model performance, with categorical_crossentropy generally outperforming mean_squared_error across different hyperparameter settings. Lastly, the batch size appears to have a minor effect on model performance. While there are slight fluctuations in average accuracy between batch sizes of 32 and 64, the differences are not as evident as those observed with other hyperparameters.

The proposed ICGN algorithm is used for two classes of hand-gripping motor activity classification and validation of the ICGN is performed by using it for three classes of motor activity classification. The results have shown enhanced classification performance and reduced computational cost as compared to prior DL algorithms and combined DL algorithm concepts. The performance of the proposed ICGN algorithm is compared with excessively used DL algorithms such as LSTM and Bi-LSTM for the classification of the fNIRS signals. The classification accuracy for the two-class motor activity problems is enhanced from 84.89 ± 3.91 to 91.23 ± 1.60 when comparing the proposed ICGN with LSTM and 88.82 ± 1.96 to 91.23 ± 1.60 when compared with Bi-LSTM. The results of the algorithms are endorsed by statistical ANOVA tests, which show the significance of the proposed ICGN over LSTM and Bi-LSTM. The ANOVA test results indicate that there is a significant difference in the accuracies obtained using the proposed ICGN, LSTM, and Bi-LSTM algorithms for the two-class and three-class datasets. The ICGN algorithm can be used for the classification of sequential datasets and command generation for fNIRS-BCI applications, including robotic hand controlling, prosthetics and rehabilitation for amputees, and medical robot controlling applications.

It is important to note the limitations of this study and provide directions for further research. Even though the dataset utilized in this work was more significant than that of previous fNIRS-based aging studies, it was still too small for deep learning applications. Another limitation of this study is the use of the ICGN network cells within the bidirectional mechanism, like Bi-LSTM. The proposed mechanism in the ICGN neural network could be implemented in the forward and backward directions for better performance. Furthermore, in the future, the ICGN algorithm could be used for the application of other neuroimaging modality datasets.

## 7. Conclusions

This study is designed to improve the classification accuracy for an fNIRS-based BCI system using a DL-based ICGN algorithm. The ICGN algorithm uses contextual knowledge and gated processes to optimize classification. It works by efficiently filtering and ranking relevant information to enhance performance. Its capacity to learn complex patterns is improved by this integration, which makes it effective in a range of applications that call for precise classification. The average classification accuracy achieved by using the proposed ICGN algorithm is 91.23 ± 1.60, which is significantly (*p* < 0.025) higher than the LSTM and Bi-LSTM algorithms. The result shows improved performance for the proposed algorithm over traditional DL algorithms (LSTM and Bi-LSTM), signifying a major advancement in improving the classification accuracy of contemporary fNIRS-BCI system. 

## Figures and Tables

**Table 1:** Subject-wise classification accuracies by using LSTM, ICGN, and Bi-LSTM algorithms for the classification of 2-class hand-gripping HBO-fNIRS data.

Subject-Wise Classification Accuracies			
Subjects	LSTM (%)	ICGN (%)	Bi-LSTM (%)
Sub 1	77.75	91.21	85.31
Sub 2	84.13	91.82	88.69
Sub 3	83.03	90.63	89.46
Sub 4	80.52	86.04	86.59
Sub 5	88.95	92.00	90.45
Sub 6	85.90	91.89	91.43
Sub 7	86.15	93.27	88.71
Sub 8	83.72	90.91	88.67
Sub 9	87.58	92.00	89.97
Sub 10	90.43	91.70	91.51
Sub 11	84.39	90.28	89.56
Sub 12	86.01	90.89	88.57
Sub 13	87.77	90.63	90.54
Sub 14	82.25	93.97	87.50
Sub 15	76.27	91.15	86.26
Sub 16	90.43	91.42	91.27
Sub 17	87.59	92.33	86.72
Sub 18	80.58	89.73	85.92
Sub 19	87.64	90.89	88.36
Sub 20	86.68	92.81	91.34
**Average**	**84.89 ± 3.91**	**91.23 ± 1.60**	**88.82 ± 1.96**

**Table 2:** ANOVA test results for the 2-class dataset.

Group 1	Group 2	Mean Difference	*p*-adj	Reject Null Hypothesis
BiLSTM	LSTM	−3.953	0.0001	True
BiLSTM	Proposed ICGN algo.	2.437	0.0159	True
LSTM	Proposed ICGN algo.	6.39	<0.001	True

**Table 3:** ANOVA test results for the 3-class dataset.

Group 1	Group 2	Mean Difference	*p*-adj	Reject Null Hypothesis
BiLSTM	LSTM	−1.85	0.0034	True
BiLSTM	Proposed ICGN algo.	4.28	0.0284	True
LSTM	Proposed ICGN algo.	6.15	<0.001	True

## Data Availability

Data can be requested from the corresponding author.
